# Large scale, single-cell FRET-based glucose uptake measurements within heterogeneous populations

**DOI:** 10.1016/j.isci.2022.104023

**Published:** 2022-03-03

**Authors:** Adam J.M. Wollman, Dimitrios Kioumourtzoglou, Rebecca Ward, Gwyn W. Gould, Nia J. Bryant

**Affiliations:** 1 Department of Biology and York Institute of Biomedical Research, University of York, York YO10 5DD, UK; 2 Biosciences Institute, Newcastle University, Newcastle upon Tyne NE2 4HH, UK; 3 Strathclyde Institute of Pharmacy and Biomedical Sciences, University of Strathclyde, 161 Cathedral Street, Glasgow G4 0RE, UK

**Keywords:** Optical imaging, Biological sciences, Cell biology, Bioinformatics, Biocomputational method

## Abstract

Fluorescent biosensors are powerful tools allowing the concentration of metabolites and small molecules, and other properties such as pH and molecular crowding to be measured inside live single cells. The technology has been hampered by lack of simple software to identify cells and quantify biosensor signals in single cells. We have developed a new software package, FRETzel, to address this gap and demonstrate its use by measuring insulin-stimulated glucose uptake in individual fat cells of varying sizes for the first time. Our results support the long-standing hypothesis that larger fat cells are less sensitive to insulin than smaller ones, a finding that has important implications for the battle against type 2 diabetes. FRETzel has been optimized using the messy and crowded environment of cultured adipocytes, demonstrating its utility for quantification of FRET biosensors in a wide range of other cell types, including fibroblasts and yeast via a simple user-friendly quantitative interface.

## Highlights

• FRETzel is a new software package for easy analysis of FRET signals in cells
• FRETzel is used to quantify glucose uptake in adipocytes of different sizes
• Reduced glucose uptake suggests that larger adipocytes have lower insulin sensitivity
• FRETzel is demonstrated on a range of cell types: yeast, fibroblasts, and adipocytes

## Graphical abstract

**Figure:**
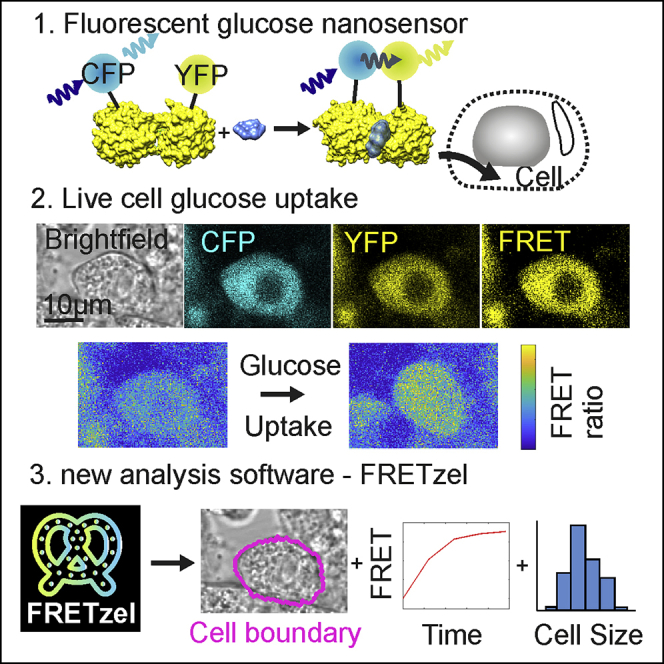

## Introduction

Cells within a population invariably exhibit significant physical and metabolic heterogeneity. This is true for both *in vitro* and *in vivo* and has important implications for research on numerous disease states including diabetes and cancer (Altschuler and Wu, 2010; Shackleton et al., 2009). For example, studies in humans indicate a relationship between fat cell (adipocyte) size and the degree of insulin sensitivity (Stenkula and Erlanson-Albertsson, 2018); nonobese individuals with Type-2 diabetes have larger adipocytes than healthy controls (Acosta et al., 2016; Verboven et al., 2018). Human adipocytes range in size from ∼70 to >270 μm in diameter, with small and large cells thought to have different metabolic properties (Acosta et al., 2016; Verboven et al., 2018). Fat biopsies containing adipocytes with a larger average diameter are significantly more insulin-resistant than those with a smaller average diameter (Stenkula and Erlanson-Albertsson, 2018; Verboven et al., 2018). It is important to note, however, that samples used in these studies contained adipocytes with a wide range of cell diameters and the conclusions rely on ensemble average measurements because of technical difficulties in analyzing metabolic states at the single-cell level. Hence, there is an unmet need to examine metabolic properties of heterogeneous cellular populations.

Insulin resistance, a hallmark of Type-2 diabetes, is defined by a reduced ability of insulin to promote glucose uptake into fat and muscle (Petersen and Shulman, 2018). Glucose uptake is routinely measured using radiolabeled glucose analogs (Manchester et al., 1990). Although powerful, this approach is limited to population-level analyses and does not allow changes to be monitored at the single-cell level. More recently, glucose-responsive FRET-sensors have been used to examine glucose uptake in single cells within a population (Kovacic et al., 2011). These FRET-sensors have proven useful for imaging glucose flux in mammalian cell lines (Takanaga et al., 2008) and investigating the dynamics of intracellular glucose regulation in a variety of human and mammalian cell lines (John et al., 2008). Genetically encoded FRET-glucose nanosensors have also been used to dissect the dynamics of cytosolic glucose levels in 3T3-L1 fibroblasts/adipocytes and CHO cells (Chowdhury et al., 2014; John et al., 2011; Kovacic et al., 2011). FRET-based glucose sensors have also proved instrumental in identifying the effects of potential therapeutics on glucose transport and hexokinase activity in cancer cells (Ghezzi et al., 2019). Fluorescent glucose analogs, 2-NBDG and 6-NBDG, have also been used to measure glucose uptake but have recently been shown to enter cells using altered transport mechanisms compared to glucose, thus raising concerns regarding their use in these measurements (Hamilton et al., 2021).

These studies represent an exciting development with the potential to dissect complexities within populations. However, studies using these sensors are limited by the inability to monitor multiple cells within a population simultaneously, necessitating labor-intensive analysis and time-consuming experimentation. Existing software packages for FRET quantification are either region of interest (ROI)-based (Roszik et al., 2008) or rely on fluorescent signals to identify cells (Kim et al., 2017); however,ROI methods cannot identify individual cells, and using fluorescence to identify cells is unreliable, at best, in heterogeneous populations. Other general-purpose cell segmentation and analysis softwares are often complex and require membrane staining or high contrast membranes (Table 1). We have developed a software package (FRETzel) to simplify fluorescence based single-cell glucose uptake measurements in heterogeneous cell populations. Using easily acquired confocal or epifluorescence microscopy data obtained from cells expressing a glucose FRET nanosensor, our software allows the user to specify cells of interest in a field of view, then defines the pixels belonging to each specified cell, and calculates the FRET ratio for each cell as a function of time. The pixel segmentation is either based on active contouring of an initial user-specified circular guess or uses thresholding combined with watershedding; therefore, it performs extremely well in dense, heterogeneous cell populations – common features of differentiated cell lines in culture. Our approach will be of wide general applicability in studies of complex cell systems and can be adapted for any metabolite that can be assayed using an FRET-sensor.

**Table 1:** Summary of existing cell segmentation and FRET quantification software

Software	Description	ROI	Cell Segmentation	FRET/Intensity Quantification	Reference
FRETzel	Cell segmentation through circular active contouring, including cell and ROI intensity quantification	Yes	Yes	yes	This study
CellX	Cell segmentation using membrane patterns, includes some intensity quantification	No	failed on adipocytes	yes	(Mayer et al., 2013)
CellProfiler	Open platform for cell image analysis, which supports manual segmentation and intensity quantification through pipeline development	Yes	yes - but only by manual drawing of cell boundaries	yes	(McQuin et al., 2018)
CellSegm	Membrane stain based cell segmentation	No	requires membrane staining	no	(Hodneland et al., 2013)
OMAL Toolbox	MATLAB toolbox for cell image analysis	No	requires fluorescence	no	(Baggett et al., 2005)
AccPbFRET	FRET quantification toolbox	Yes	No	yes	(Roszik et al., 2008)
FLIM-FRET analyzer	FRET quantification toolbox with fluorescence based cell segmentation	Yes	requires fluorescence	yes	(Kim et al., 2017)

This includes whether they support region of interest (ROI) based quantification, cell segmentation, and intensity quantification.

## Results

### FRETzel allows quantification of FRET in adipocytes

**Figure 1:**
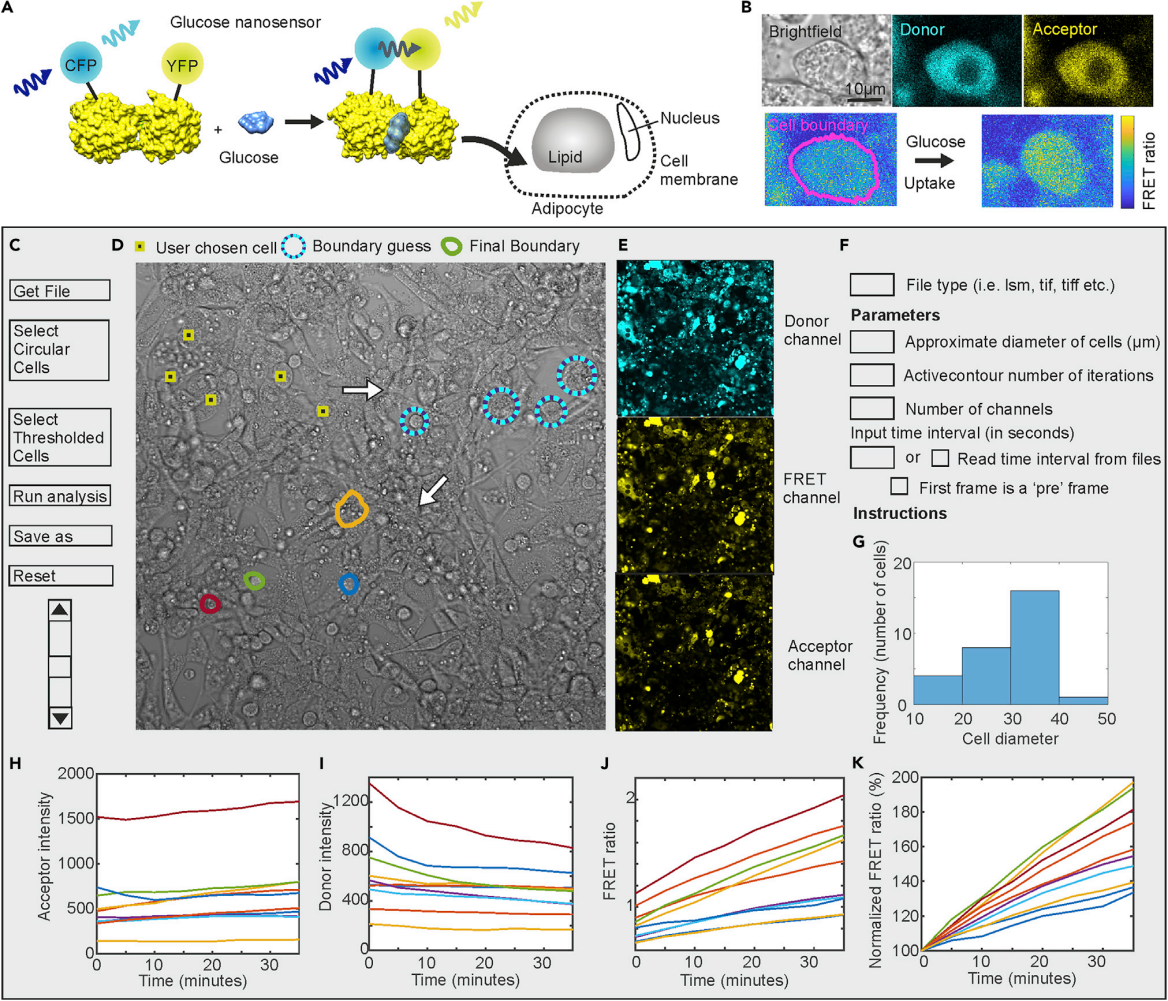
Glucose nanosensors and FRETzel (A) Schematic of glucose nanosensor operating in 3T3-L1 adipocytes. Glucose binding induces a conformational change in the glucose-binding protein, bringing the fluorophores into a closer proximity allowing FRET. (B) Confocal microscopy images of 3T3-L1 adipocytes expressing the sensor. Brightfield generated from the transmitted part of the donor excitation is shown in gray, the donor signal in cyan, and the acceptor in yellow. The pixel-by-pixel FRET ratio is shown below using a parula colormap, with the cell boundary shown in pink. Glucose uptake causes an increase in FRET values. C-K. Mock-up of the software user interface. (C) Buttons for basic functions. (D) Transmission confocal image of adipocytes with segmentation steps shown. User first choses cells for analysis (yellow/black squares) and the software places a circle (blue/cyan) of set guess diameter. User then adjusts the circle to approximately match the cell boundary, before the software uses active contouring to define the final cell boundary (colored outlines). (E) Corresponding fluorescence channels: direct donor excitation (cyan), donor excited acceptor (yellow), and direct acceptor excitation (yellow). (F) User input parameters. (G) Histogram distribution of cell diameter (microns). (H–K). The acceptor (H) and donor (I) intensity, FRET ratio (J) and FRET ratio normalized to the initial value (100%) (K) as a function of time after adding 25mM glucose to glucose starved adipocytes (different colored lines each represent data collected from single cells within the same field contemporaneously).

The glucose sensor-FLII12Pglu-700uDelta6 comprised of the *E.coli* glucose binding protein (PDB 2FW0) modified with a cyan (CFP) and yellow (Citrine) fluorophores(Takanaga et al., 2008) was introduced into 3T3-L1 adipocytes, a well-characterized and widely used model system to study insulin-stimulated glucose transport. This advanced and well-characterized sensor is specific for both and high sensitivity to glucose and robustness toward environmental changes such as pH (Takanaga et al., 2008) Using this system, we first demonstrated that our software could identify single 3T3-L1 adipocytes and record glucose-dependent and time-dependent increases in FRET signal (Figures 1 and S1). We set out to benchmark our software against the other two freely available programs that could potentially be used to identify cells and quantify FRET, CellX, and CellProfiler (Table 1); however, CellX could not identify adipocytes because of their low contrast membranes and CellProfiler required completely manual segmentation (Figure S2). Thus, FRETzel is the first software package, to our knowledge, allowing quantification of cell morphology and FRET-based glucose uptake in adipocytes.

### Larger adipocytes are less insulin responsive than smaller adipocytes

To demonstrate the utility of our software, we used it to test the hypothesis that smaller fat cells are more insulin responsive than bigger ones (Stenkula and Erlanson-Albertsson, 2018). Previous studies addressing the question are limited by a requirement to assay transport on populations of cells of different average sizes (Cotillard et al., 2014; Small et al., 2018; Zhang et al., 2017). The key advantage of the software we have developed is that it offers the ability to examine glucose transport in a large number of single cells within a heterogeneous population and hence allows to test this hypothesis directly.

**Figure 2:**
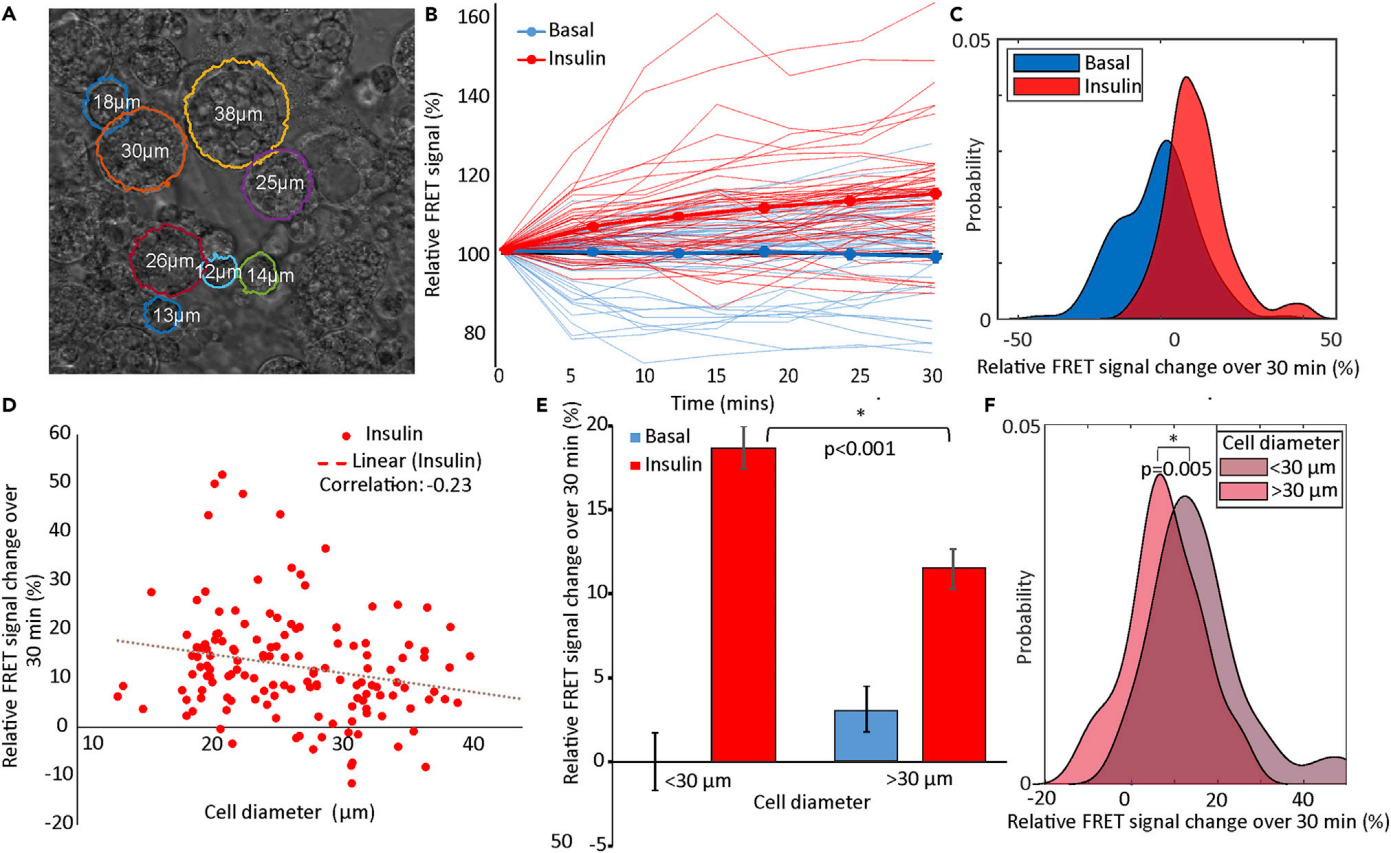
Insulin sensitivity and adipocyte size (A) Transmission confocal micrograph of 3T3-L1 adipocytes with software segmented boundaries (overlaid colored lines) and measured diameters shown. (B) Relative FRET signal, normalized to starting value, as a function of time of serum starved adipocytes treated with 1μM insulin (red lines) or vehicle (blue lines) as control for basal condition. Thin lines show individual cells and thick show the population mean (N = 140 cells from five independent replicates). (C) Distribution of relative FRET change over 30 min for each adipocyte in B generated as a kernel density estimate. (D) Relative FRET change over 30 min for each insulin stimulated adipocyte in B as a function of cell diameter. Dotted lines show free linear fits, with correlation coefficients in insert. (E) The mean total relative FRET change comparing basal and 30 min insulin treatment of adipocytes with diameters below or above the mean cell diameter of ∼30 μm, normalized to small basal cells. Error bars showing SE and ∗ indicating significant differences to p < 0.05 using Student's *t*-test. (F) Distribution of relative FRET change over 30 min for each insulin stimulated adipocyte in B generated as a kernel density estimate, separated by adipocyte diameter. ∗ indicating significant differences to p < 0.05 using Kolmogorov-Smirnov test. Compared to the initial FRET ratios (Figure S5B), only after insulin stimulation is there a difference between small and large adipocytes suggesting there is no bias between the two groups based on expression levels.

In Figure 2 we show such an analysis of differentiated 3T3-L1 adipocytes expressing the glucose sensor-containing cells with a range of diameters (Figure S3). Insulin was added at time zero, and FRET signals recorded from multiple samples and fields of view containing approximately 140 cells. Images were taken at 5 min intervals, for 30 min, limited only by the speed of the confocal microscope and the photostability of the dyes (Takanaga et al., 2008). Time course images were then analyzed by using the software (Figure 2). Each time series was loaded before clicking on individual cells to be analyzed, identified by morphology, and presence of fluorescent signal. After adjusting the input circular boundary guess, cell boundaries were computationally generated by FRETzel using active contouring, and the mean cellular intensity was calculated for each channel. FRETzel segmentation (Figure 2A) was compared to manual segmentation (Figure S4) using the Jaccard similarity coefficient (also known as intersection over union), which varies between 0 and 1 with 1 being a perfect match. FRETzel segmentation agreed well, with a mean score of 0.76 ± 0.02. The ability to segment the data by cell diameter allows comparing the effect of insulin on smaller and larger adipocytes.

Insulin-stimulated cells showed a clear increase in relative FRET signal over time compared to basal cells treated with media as a control (Figure 2B). Relative FRET change over the 30 min of the experiment was positive for insulin treated cells and distributed around zero for basal cells with a high variance (Figure 2C). Plotted against cell diameter (Figure 2D), we measured a small negative correlation between adipocyte diameter and relative FRET change of −0.23 under insulin stimulation, with no correlation in the basal cells (Figure S5A). Pooling adipocytes into small and large bins based on mean cell diameter resulted in no significant differences in initial FRET values (Figure S5B); however, it demonstrated a significant difference between small and large adipocytes under insulin stimulation (Figures 2E, 2F, and S5C), with larger cells producing lower relative FRET response, consistent with reduced insulin-stimulated glucose transport. By fitting FRET vs time traces (Figure 2B) with simple exponentials (Figure S6A), we could also compare the rate constant for stimulation of transport by insulin between individual cells. This rate was independent of cell size (Figure S6B), even when pooled into small and large diameter bins (Figure S6C). This is important as it indicates that the difference in insulin response in larger adipocytes is not simply because of larger cells taking longer to reach their maximally stimulated state.

### FRETzel can be used to measure FRET in diverse cell types

**Figure 3:**
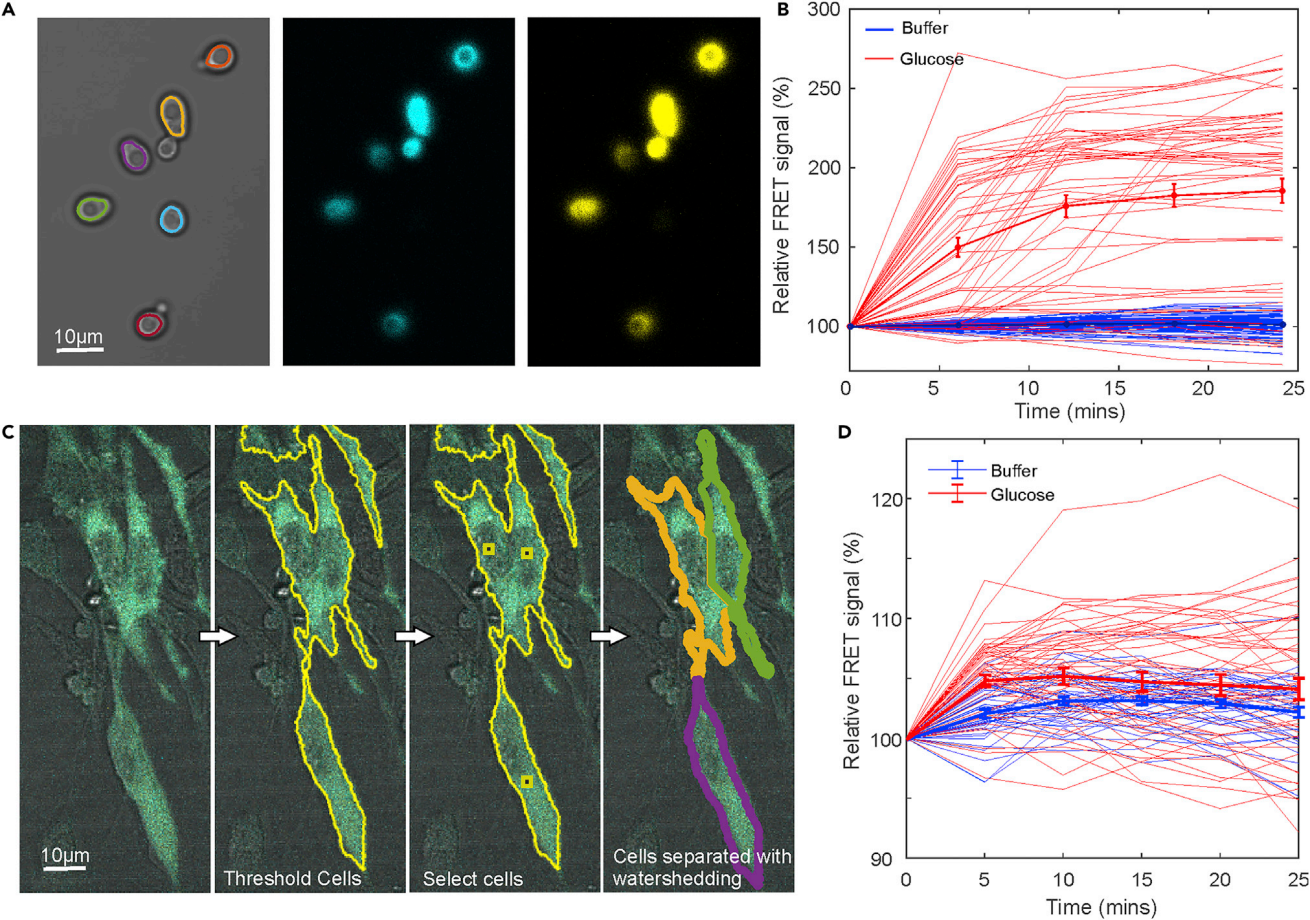
FRETzel applied to other cell types (A) Budding yeast in transmission brightfield with cell segmentation indicated as colored lines with fluorescence images of donor (cyan) and donor excited acceptor (yellow). (B) Normalized FRET ratio as a function of time for glucose starved yeast cells exposed to 2%(w/v)glucose or control media containing no glucose for a population of N = 50 cells. Thick lines indicate the mean with error bars representing the SE of the mean. (C) Composite image of murine fibroblasts expressing the glucose sensor with brightfield in gray and sensor fluorescence in cyan. Segmentation indicated schematically in yellow outline: fluorescence is thresholded by the user to separate cells from background, cells of interest are selected, and a watershedding algorithm separates individual cells. (D) Normalized FRET ratio as a function of time for glucose starved fibroblasts exposed to 2%(w/v)glucose or control media containing no glucose for a population of N = 50 cells. Thick lines indicate the mean with error bars representing the SE of the mean.

As further proof-of-concept, we also tested our software by measuring glucose uptake in another round cell type, budding yeast (Figures 3 and S7). FRETzel was able to segment yeast cells well (Figures S3A and S7) and measure a high relative FRET signal increase in glucose-starved yeast cells expressing the sensor and exposure to glucose compared to control cells (Figure 3B).

We also implemented additional software tools to facilitate the analysis of more complex cell shapes, such as undifferentiated mammalian fibroblast-like cell lines, which adopt a more ‘spread’ morphology in culture. Our additional tools use the fluorescence image and a user-set threshold via a slider (Figure 1C) to define cell regions from the background. The user then clicks on desired cells within regions and the software uses a distance-based watershedding algorithm to separate individual cells (Figure 3C). We used these tools to also measure the glucose uptake in murine fibroblasts and confirmed a glucose-dependent increase in FRET as expected (Figure 3D). This validates FRETzel as a suitable tool for a wide array of cell types.

## Discussion

We validated our software by applying it to insulin response in fat cells. Increases in fat deposits can be mediated by enlargement (hypertrophy) of existing cells and/or increased adipocyte numbers (hyperplasia) (Scherer, 2019). Numerous studies have revealed that adipocytes from patients with Type 2 diabetes exhibit hypertrophy (Ghaben and Scherer, 2019). Consistent with this notion, separation of human adipocytes — taken from fat biopsy material — into small (81.3 ± 14μm) and large (114.0 ± 17μm) populations indicates that larger cells exhibit reduced insulin-stimulated translocation of the facilitative glucose transporter GLUT4 to the cell surface (Franck et al., 2007). Unfortunately, this interesting study was unable to measure glucose transport into adipocytes of different sizes. Our results have taken the finding of this study to the next level by collecting data from individual cells within a heterogeneous population, thereby supporting the hypothesis that larger fat cells exhibit reduced insulin-stimulated glucose transport, as demonstrated by the lower increase in FRET signal in larger cells compared to smaller cells (Figure 2E). It is important to note that this difference is not simply because of larger cells taking longer to reach their maximally stimulated state: another key advantage of our approach is the ability to extract dynamic information, which is not possible to obtain by measuring glucose uptake using radiolabeled analogs. By fitting FRET vs time traces with simple exponentials, it is possible to compare the rate constant for stimulation of transport by insulin between individual cells (Figure S6), which we found to be independent of cell size.

We used the ability to assay glucose transport in single cells within a population to demonstrate the utility of our platform. Our work paves the way for single-cell metabolic analyses in multiple systems, including primary adipose tissue, cancer cells, and stem cells (Altschuler and Wu, 2010; Shackleton et al., 2009). This advancementt will be particularly important because of the high levels of heterogeneity that characterizes the complex tissues and tumors and is known to underscore both the complexity of tissue responses (e.g., to insulin as shown here) and their response to therapeutics (Cao, 2019; Suchacki et al., 2020). It is important to note that this platform is not limited to measuring glucose uptake but can be used in conjunction with any FRET biosensor, in any cell line, such as those for cAMP (Paramonov et al., 2015). FRET-based biosensors are becoming increasingly available to detect changes in a wide variety of small molecules and ions (Liu et al., 2017)- FRETzel provides the cell biology community with an ideal platform that could be used alongside these reagents to address an almost limitless number of fundamental biological questions in their preferred cell type with a simple and user-friendly interface.

### Limitations of the study

Fluorescent biosensors represent an exciting set of tools to measure metabolites and other chemical signatures in living cells; however their adoption has been held back by the lack of software tools to analyze the data. We developed FRETzel to address the need for a simple program to robustly identify cells in heterogeneous populations and quantify fluorescent signals. Although many advances have been made in fully-automated cell segmentation softwares, particularly with machine learning (Ounkomol et al., 2018), these packages still require large training data sets, programming expertise from the user, and perform poorly in messier images. For this reason, we designed FRETzel to utilize a semi-automated approach, thereby simplifying and speeding up analysis while maintaining user input and validation. We believe FRETzel is an excellent tool to generate data sets to train machine learning models and its outputs could readily be used with new, easy-to-use machine learning tools such as ZeroCostDL4Mic (von Chamier et al., 2021). Future versions of FRETzel could also interface with deep learning algorithms directly.

We also focused on FRETzel for use with FRET biosensors which requires calculation of only a simple ‘raw’ FRET ratio (donor excited acceptor intensity/donor intensity). True FRET ratio requires the calculation of correction factors to account for spectral bleed-through of the dyes. These corrections need separate control experiments of further fluorescent cell constructs exhibiting no FRET and there is no single consensus method for their calculation (Hochreiter et al., 2019). FRET biosensors do not require corrected FRET to observe changes in signal and the complexity around FRET calculations may have held back biosensor adoption. Therefore, FRETzel uses simple FRET calculations by default but could also be used to calculate corrected FRET by using it to analyze control data and applying the correction factors. For example FRETN (Xia and Liu, 2001) could be calculated by applying FRETzel to cells expressing only the donor or acceptor fluorophores, measuring their bleedthrough.

The FRET sensor used here has been very thoroughly tested (Takanaga et al., 2008), including in HepG2 cells exposed to varying concentrations of glucose and the cytoplasmic accumulation rate as a function of extracellular glucose fit with Michaelis-Menten equations. These fits are comparable to GLUT1 affinity to d-glucose on the plasma membrane. We further characterized the performance of the sensor in adipocytes by similarly exposing adipocytes to different concentrations of extracellular glucose. We also found a proportionate response (Figure S1), consistent with the idea that our sensor is measuring elevated cytosolic glucose levels, as a result of increased glucose transport. It is not possible to relate FRET signal to glucose metabolism using this system, as we have no means to measure glucose metabolism and only capture cytosolic glucose levels. Most studies agree that transport across the plasma membrane is the rate-limiting step for glucose metabolism in adipocytes, and thus elevations in cytosolic glucose, as measured here, likely reflect increased rates of glucose transport (Hjøllund and Pedersen, 1988).

## STAR★Methods

### Key resources table

**Table:** 

REAGENT or RESOURCE	SOURCE	IDENTIFIER
**Chemicals, peptides, and recombinant proteins**		

Geneticin	Gibcom	10131035
3-isobutyl-1-methyxanthine	Sigma-Aldrich	I5879
Dexamethasone	Sigma-Aldrich	D1756
Troglitazone	Cayman chemicals	71740
human recombinant insulin	Sigma-Aldrich	I9278
concanavalin-A	Merck	234567M

**Experimental models: Cell lines**		

3T3 L1	ATCC	ATCC CL-173

**Experimental models: Organisms/strains**		

*S. cerevisiae* BY4741	University of Gothenburg	N/A

**Recombinant DNA**		

Plasmid pcDNA3.1 FLII12Pglu-700uDelta6	Takanaga et al. (2008)	Addgene #17866

**Software and algorithms**		

FRETzel	This paper	https://doi.org/10.5281/zenodo.5713684
MATLAB	Mathworks	NA

**Other**		

Dulbecco’s modified eagle medium (DMEM)	Gibco	#11960044

### Resource availability

#### Lead contact

Further information and requests for resources and reagents should be directed to and will be fulfilled by the lead contact, Adam Wollman (adam.wollman@newcastle.ac.uk).

#### Materials availability

This study did not generate new unique reagents.

### Experimental model

#### Cell lines and cell culture

3T3-L1 murine fibroblast cells were cultured in 3T3-L1 fibroblast growth media at 37°C under 5% CO_2_ concentration.

### Method details

#### Cell growth media

All cell culture media were filter sterilized through a 0.2 μm pore size filter.

• 3T3-L1 fibroblast growth medium: Dulbecco’s modified eagle medium (DMEM) high glucose, no glutamine medium, (Gibco #11960044) supplemented with 10%(v/v) new-born calf serum (NCS) (Gibco #11580506) and 1% GlutaMAX (x100 Gibco #11574466).
• 3T3-L1 fibroblast selection medium: 3T3-L1 fibroblast growth medium supplemented with 0.5mM geneticin (Gibcom #10131035).
• 3T3-L1 adipocyte growth medium: DMEM high glucose, no glutamine supplemented with 10% (v/v) foetal bovine serum (FCS) (Gibco #10082147), 1% GlutaMAX and 0.5mM geneticin.
• 3T3-L1 differentiation medium 1: 3T3-L1 adipocyte growth media supplemented with 0.5mM 3-isobutyl-1-methyxanthine (IBMX) (Sigma-Aldrich #I5879), 0.25mM dexamethasone (Sigma-Aldrich #D1756), 5μM troglitazone (Cayman chemicals #71740) and 1 μg/mL (170 nM) human recombinant insulin (Sigma-Aldrich #I9278).
• 3T3-L1 differentiation medium 2: 3T3-L1 adipocyte growth media supplemented with 1 μg/mL (170 nM) human recombinant insulin.

#### Production of 3T3-L1 cell lines stably expressing FLII12Pglu-700uDelta6 glucose sensor

The FLII12Pglu-700uDelta6 sensor has been shown to respond to glucose with comparative affinity to GLUT1 on the plasma membrane and so closely reflect actual transport of glucose into the cell(Takanaga et al., 2008). 3T3-L1 fibroblast cells were grown on six well plates until 60% confluency. Cells were transfected with pcDNA3.1 FLII12Pglu-700uDelta6 (Addgene #17866) using XFectTM transfection reagent (TaKaRa #631318) according to the manufacturer’s instructions. Briefly, 5μg of Plasmid DNA were mixed with 1.5μL Xfect polymer and reaction buffer up to a volume of 100μL, vortexed and incubated at room temperature for 10min. The whole volume of mixture was added drop wise into each well containing 100μL of 3T3-L1 fibroblast growth medium and incubated at 37°C 4 h. Medium was subsequently removed and replaced with 2mL of 3T3-L1 fibroblast growth medium. Plates were then incubated for 48 h. Subsequently, the medium was removed and replaced with 3T3-L1 fibroblast selection medium for at least two weeks. During the selection period all untransfected cells died and colonies of 3T3-L1 fibroblast cells stably expressing FLII12Pglu-700uDelta6 glucose sensor appeared (usually 2-3 colonies per well). Colonies of each well were expanded and used for differentiation into adipocytes.

#### Differentiation of 3T3-L1 murine fibroblasts into adipocytes

3T3-L1 cells that stably express FLII12Pglu-700uDelta6 glucose sensor were grown on 4-chamber coverslip slides (Nunc^TM^ Labtech^TM^ #155383) containing 3T3-L1 fibroblast selection medium to confluency. On the day of differentiation cells were washed with serum free DMEM and differentiation medium 1 was added (500μL per chamber). After three days the medium was replaced by differentiation medium 2 (500μL per well) and after two days cells were fed with adipocyte growth media (replacing the media every other day until the adipocytes were used for experiments - typically on the 8th-12th day after the initiation of differentiation).

Insulin stimulation: adipocytes were incubated for 2h in serum free no phenol red DMEM (Gibco #31053028). Cells were then stimulated by addition of 1μM insulin (final concertation) or not (basal controls-addition of equivalent volume of PBS) as shown in the figures.

#### Confocal microscopy

Images of adipocytes expressing the glucose sensor were taken using a Zeiss LSM 780 confocal microscope with laser lines at 458nm for CFP excitation and 514nm for YFP excitation. A 20x plan-apochromat NA = 0.8 objective lens was used to generate a 571 × 571μm field of view at 210 nm/pixel. Pixel intensity was digitised into 12 bits with a scan speed taking ∼5 min per image. Laser power and gain were set to maximise signal without saturation and avoid photobleaching. Brightfield images were recorded from the transmitted light from the 514nm channel. All settings were kept constant across all experiments. Images were acquired using Zeiss ZEN10 software.

#### Software

Bespoke image analysis software was written in MATLAB™. A full software manual is provided with the software in the DOI in the key resources table. Image data is read using the Bio-formats package from the Open Microscopy Environment (Linkert et al., 2010) in any compatible file format. Images must be formatted as xyct stacks, with channels ordered as brightfield, donor, acceptor and direct acceptor. Each time point can be in the same stack or in separate files. Cells can be defined by the user by clicking roughly on the centre of each one. The software then places a circle of set diameter, defined by the user settings, on each cell. The user can then adjusts the diameter of each circle, including separately horizontally and vertically to create an ellipse, to approximately match the boundary of the cell. Once complete for all cells chosen by the user, the cell boundary is determined by active contouring. This algorithm, also known as ‘snakes’ (Kass et al., 1988) uses a deformable spline to find gradients and contours in an image by minimising energy. The user can set how many iterations of contouring take place and thus how far the input ellipse will deform. Alternatively, the user can set an intensity threshold using the slider to segment cells from background by threshold. The user can then separate cells by clicking on those chosen before a watershed algorithm is used to define cell boundaries. This algorithm uses the binary distance transform from the centre of each click and finds the ‘ridges’ at the boundary between each cell. Once the user is satisfied with the boundaries, the software measures the mean pixel intensity inside the boundary for each cell in each channel and over time. The FRET ratio is simply calculated as the acceptor intensity divided by the donor which, although not the true or corrected FRET ratio, is all that is needed for biosensor quantification. Output analysis can then be saved in a user specified folder. This output includes the intensity and FRET values as a function of time in comma separated values (csv) format and an image of the cell boundaries.

#### Comparison with other software

Of the other cell image and FRET analysis software found (Table 1), CellX and CellProfiler were potentially suitable to analyse glucose sensor expressing adipocytes. A sub-region of representative transmission brightfield data was chosen and segmented with FRETzel (Figure S2A). The same image was loaded into CellX, minimum and maximum seed sizes set based on the smallest and largest visible adipocytes respectively. The membrane signal was trained on 5 cells with ∼8 line profiles per cell. CellX successfully identified 3 cells but was unable to determine their boundaries due to poor contrast in the membrane. The same image was loaded into CellProfiler. All built in segmentation tools were intensity based, leaving only manual segmentation. It should be noted that CellProfiler is an analysis platform allowing the development of analysis ‘pipelines’ requiring significant development (and associated expertise) from the user.

#### Data analysis

Confocal microscopy images were analysed using the bespoke software. Adipocyte cells were chosen based on their morphology in brightfield, including a rounded shape and visible internal fat deposits, as well as the presence of fluorescent signal in donor and acceptor channels. Around 10 active contour iterations were used to find the boundary of ∼50 differentiated adipocytes in each image. Normalized FRET ratios were calculated outside of the software by dividing by the first value and the total relative change over time calculated by subtracting the final point from the first. This is a readout of the magnitude of the glucose concentration change in each cell in response to insulin For dynamic analysis each normalized FRET vs time trace was fitted with an exponential of the form:

$$F\left(t\right)=F_{final}(1-\exp\left(-k_{u}t\right))$$

Where F(t) = FRET ratio as a function of time (t), F_final_ = final normalized FRET ratio. k_u_ = uptake rate. Poor fits as determined by the coefficient of determination, R^2^ lower than 0.8 were discarded. This rate characterizes how quickly cells respond to insulin and uptake glucose.

#### Yeast experiments

*S. cerevisiae,* BY4741 wild type was transformed with plasmid pDR-GW FLII12Pglu-700μδ6 (Bermejo et al., 2010) from AddGene using standard lithium acetate protocols. Cells were grown on YPD medium plates (20 g/L Bacto Peptone, 10 g/L Yeast Extract) supplemented with 4% glucose (w/v) at 30°C before overnight liquid cultures were grown in YNB media with 40 g/L glucose, sub-cultured and grown until mid-logarithmic phase, *OD*_*600*_ *∼ 0.7*. Cells were washed to remove glucose and placed in YNB without glucose for 1-2 h. Cells were transferred to concanavalin-A coated glass-bottom petri dishes (Ibidi μ-Dish 35 mm, 81158), allowed to settle for 5 min and washed with YNB media with a pipette. Prior to imaging, cells were washed with YNB containing 2% glucose or control 0% glucose. Yeast cells were imaged similarly to the adipocytes.

### Quantification and statistical analysis

Statistical details of experiments can be found in figure legends. Means were compared using the 2 sampled Students t-test, based on the standard deviation. Distributions were compared using 2 sample Kolmogorov-Smirnov tests. We used a threshold of p < 0.05 for significance.

## Data Availability

• Original data and microscopy data reported in this study will be shared by the lead contact upon request. • All original code has been deposited at Github and is publicly available as of the date of publication. DOIs are listed in the key resources table. • Any additional information required to reanalyze the data reported in this paper is available from the lead contact upon request.
