# Supplementary material for: Large scale, single-cell FRET-based glucose uptake measurements within heterogeneous populations
Source: iScience. 2022 Mar 3;25(4):104023. doi: 10.1016/j.isci.2022.104023 (PMC8933717; doi:10.1016/j.isci.2022.104023)
Supplement: Document S1. Figures S1–S7 [file mmc1.pdf]

iScience, Volume *25*

Supplemental information

Large scale, single-cell FRET-based glucose uptake measurements within heterogeneous populations

Adam J.M. Wollman, Dimitrios Kioumourtzoglou, Rebecca Ward, Gwyn W. Gould, and Nia J. Bryant

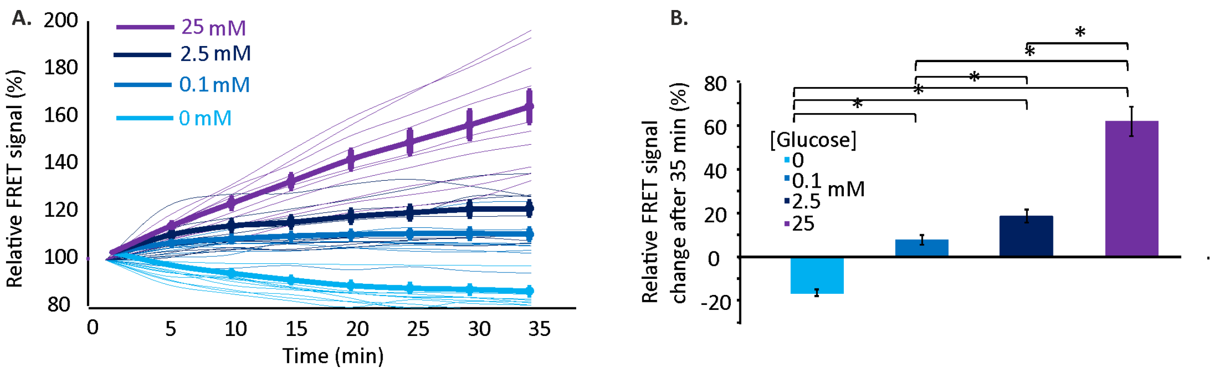

Figure S1. Characterisation of glucose sensor FRET in adipocytes exposed to different glucose concentrations, related to STAR methods. A. FRET signal as a function of time, upon incubation of glucose-starved 3T3-L1 adipocytes with increasing extracellular glucose concentrations (normalised to starting value). Thin lines show individual cells and thick lines show the population mean (N=30 cells/glucose concentration). **B**. The mean total relative FRET change over 35mins for each glucose concentration. Error bars showing standard error and * indicating significant differences to $p<0.05$ using Student's t-test.

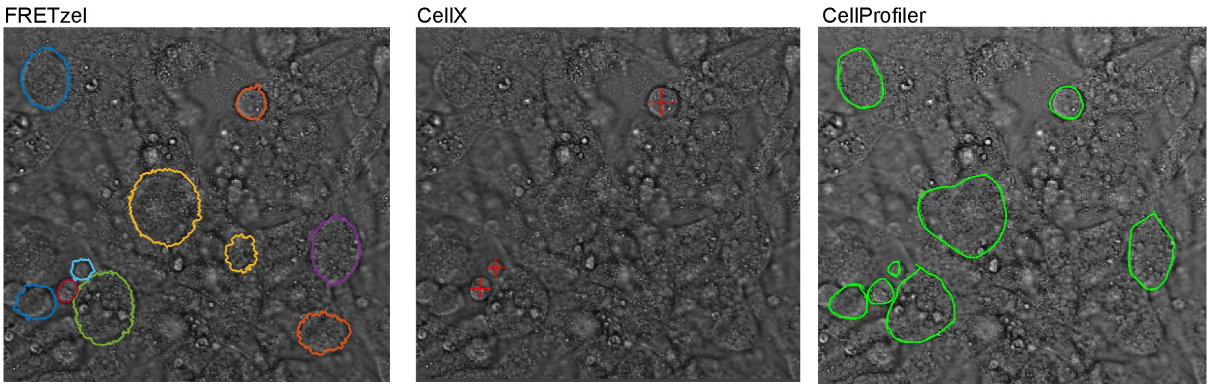

Figure S2. Comparison of adipocyte segmentation using FRETzel, CellX and CellProfiler respectively, related to Table 1. FRETzel segmented 9 cells. CellX identified 3 cells but was unable to define their boundaries due to the low contrast membrane of adipocytes. Cell profiler supported only manual segmentation.

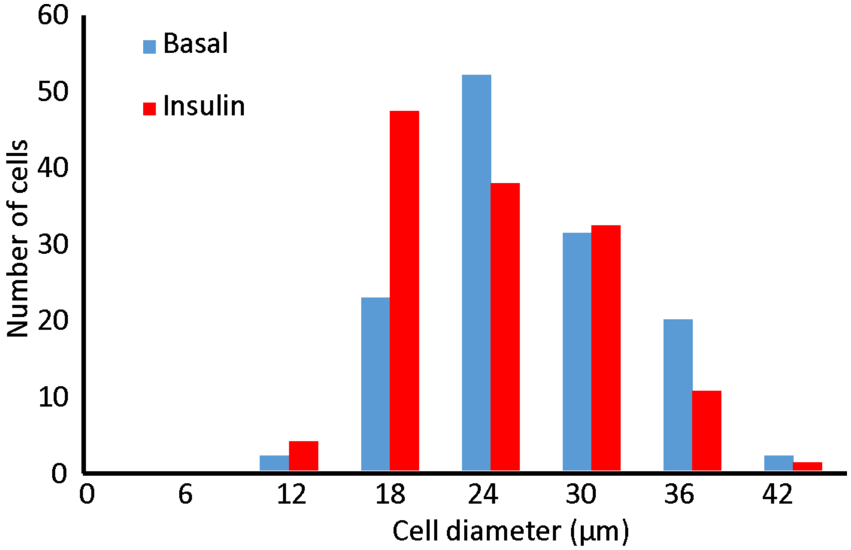

Figure. S3. Histogram of software segmented cell diameters related to Figure 2. Bin width 6 µm.

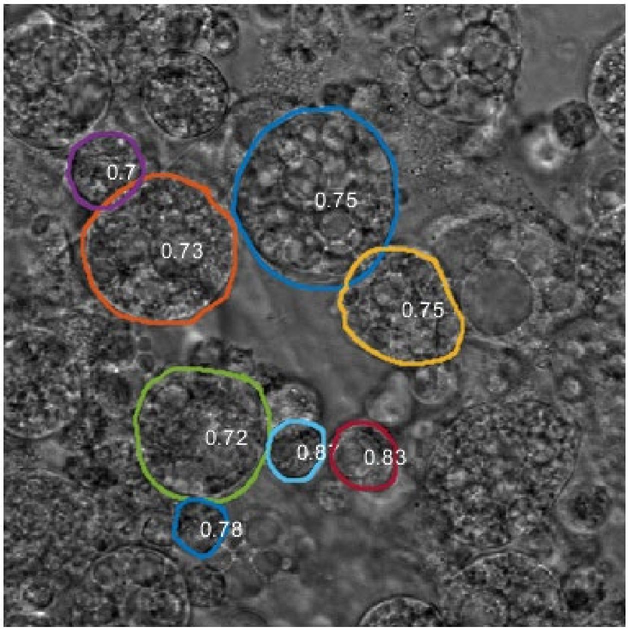

Figure S4. Manually segmented adipocytes, related to Figure 2. Shown as coloured lines overlaid on the corresponding brightfield image. The Jaccard similarity coefficient for each cell compared with FRETzel segmentation indicated.

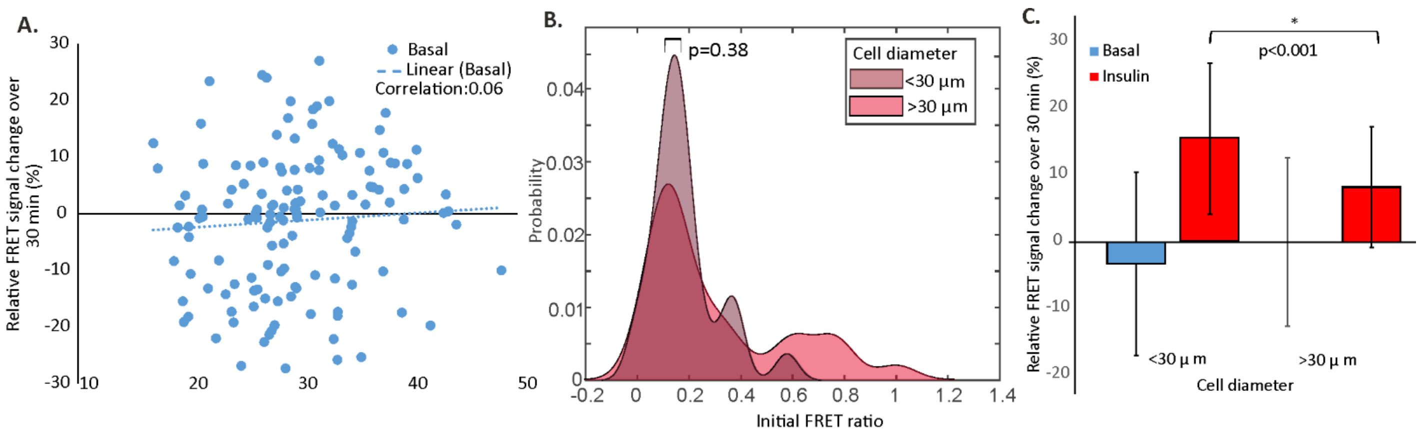

Figure S5. Adpocyte FRET characterization, related to Figure 2. A. Relative FRET change over 30 mins for each basal adipocyte in Figure 2B as a function of cell diameter.B. Distribution of inital FRET ratio for each insulin stimulated adipocyte in generated as a kernel density estimate, separated by adipocyte diameter. * indicating significant differences to $p<0.05$ using Kolmogorov-Smirnov test. C. The mean total relative FRET change comparing basal and 30 mins insulin treatment of adipocytes with diameters below or above the mean cell diameter of ~30 μm, normalised to small basal cells. Error bars showing standard deviation and * indicating significant differences to $p<0.05$ using Student's t-test.

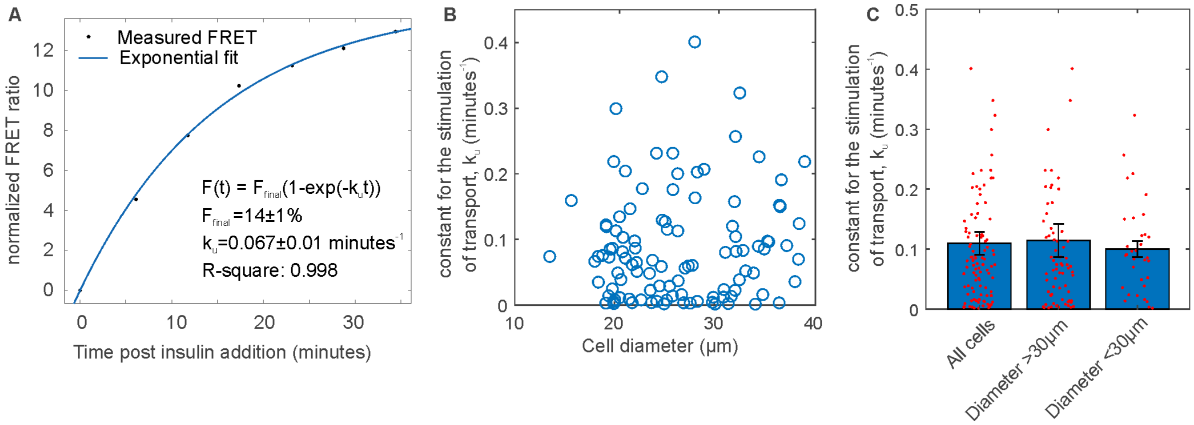

Figure S6. Characterisation of insulin stimulated glucose uptake dynamics in adipocytes, related to Figure 2. A. example exponential fit to a single cell FRET as a function of time. F(t)=FRET ratio as a function of time (t), F_{final}=final FRET ratio. k_u= rate constant for the stimulation of glucose uptake by insulin. B. The rate-constant for the stimulation of transport, k_u, as a function of size for each fitted cell with an R^2>0.8. C. The mean ± standard error of k_u, across all cells and below or above 30µm diameter with all cells shown as jittered dots. No significant differences between different cell sizes below p=0.3. N=102.

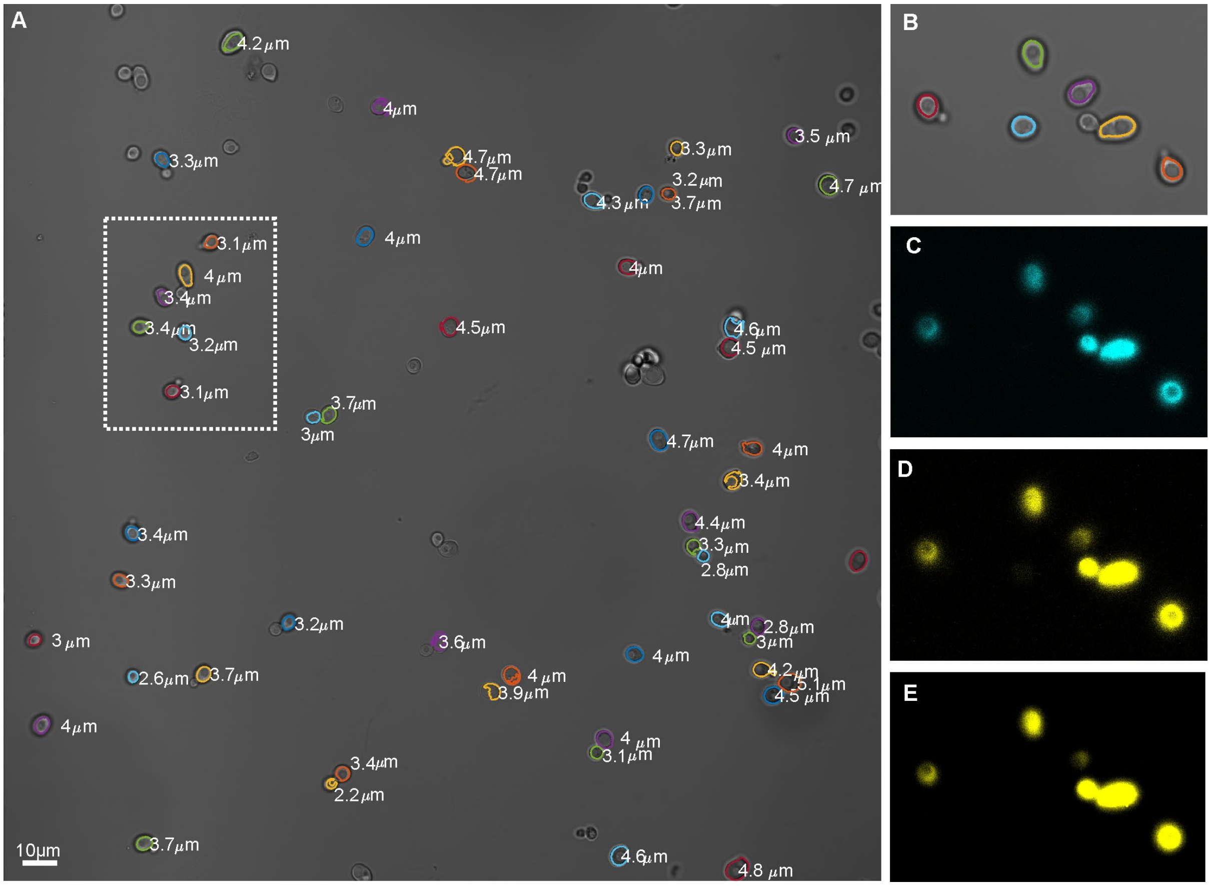

Figure S7. Segmentation of budding yeast expressing the glucose sensor, related to Figure 3. A. large field of view, 500x500µm of budding yeast in transmission brightfield with cell segmentation indicated as coloured lines and cell diameter in white. B. Zoom in of section highlighted with white dotted lines in A. C-E fluorescence images of donor, donor excited acceptor and direct acceptor excitation respectively.
